# Is a Combination of a GnRH Agonist and Recombinant Growth Hormone an Effective Treatment to Increase the Final Adult Height of Girls with Precocious or Early Puberty?

**DOI:** 10.1155/2018/1708650

**Published:** 2018-12-30

**Authors:** Wei Song, Fei Zhao, Shuang Liang, Guimei Li, Jiang Xue

**Affiliations:** ^1^Department of Pediatrics, The Second Hospital of Shandong University, Jinan, Shandong Province, China; ^2^Department of Pediatrics, Shandong Provincial Hospital Affiliated to Shandong University, Jinan, Shandong Province, China

## Abstract

The aim of treatment for idiopathic central precocious puberty (ICPP) is to increase final adult stature, for which gonadotropin-releasing hormone agonist (GnRHa) is the gold standard. Early puberty is frequently similar to ICPP, with pubertal onset only slightly advanced. Short stature may result from early pubertal onset. Some studies have suggested that recombinant human growth hormone (rhGH) should be combined with a GnRHa to improve adult height, while others have not. Here, the aim was to compare the efficacy of combined GnRHa and rhGH treatment with GnRHa or rhGH treatment alone, or no therapy, for the improvement of the final height of girls with ICPP or early puberty. Electronic databases of randomized and quasi-randomized controlled trials, in which the efficacy of GnRHa preparations was compared with that of rhGH for the treatment of children with precocious or early puberty, were searched and a meta-analysis conducted. Five studies of early puberty and four studies of ICPP were identified. There were no statistically significant differences between final adult height standard deviation score and initial height standard deviation score in the treatment of early puberty (GnRHa and rhGH versus rhGH alone or no treatment). The overall analysis of the data failed to indicate any benefit of combined therapy, while individual reports suggested that in specific instances combined therapy may be beneficial in preserving or reclaiming growth potential and improving adult height.

## 1. Introduction

Idiopathic central precocious puberty (ICPP) is defined as the early onset of phenotypically appropriate secondary sexual characteristics resulting from premature activation of the hypothalamic-pituitary-gonadal axis [1]. Although there is no precise time point demarcating precocious from age-appropriate puberty, it is commonly accepted that children presenting signs of pubertal development before a chronological age (CA) of 8 years in girls and 9 years in boys, with a pubertal response to a luteinizing hormone-releasing hormone (LHRH) stimulation test, in the absence of organic abnormalities are affected by ICPP. Rapid progression of bone age (BA) commonly occurs in ICPP children and can lead to a lower final adult height (FAH) due to the premature fusion of epiphyseal growth plates [2]. Advanced progression of secondary sexual characteristics occurring in children with precocious puberty can also lead to poor social adaptability and emotional disorders [3]. Precocious puberty is currently 10 times more common in girls [4].

Gonadotropin-releasing hormone agonist (GnRHa) has been the gold standard therapy for ICPP for more than 20 years [5]. These compounds have a high affinity for the pituitary LHRH receptor and are resistant to enzymatic degradation. Thus, through continuous stimulation, they inhibit the pulsatile secretion of gonadotropin, resulting in hormonal suppression, cessation of pubertal development, and normalization of growth and skeletal maturation rates. The goal of therapy is to halt pubertal progression and delay epiphyseal maturation, to improve growth potential. The safety and efficacy of GnRHa have been established in children with ICPP [6]. However, during treatment with GnRHa, growth velocity (GV) sometimes decreases below that of prepubertal children, such that predicted adult height may be worse. This impaired growth pattern may be due to a return to a prepubertal hormonal status and a reduction in growth hormone (GH) secretion; consequently, addition of recombinant human growth hormone (rhGH) therapy was proposed to increase GV and, ultimately, adult height [7–9].

rhGH is a routine treatment for children with GH deficiency [10], and it is also used in other short stature conditions, such as small for gestational age (SGA) and idiopathic short stature (ISS). Patients with true GH deficiency treated early with rhGH may reach their target height; however, some patients with short stature treated with rhGH do not reach their target height because they go through puberty early and have advanced BA, which results in a shorter timeframe for rhGH treatment. Physicians may choose to use GnRHa to delay the progression of BA and provide more time for effective rhGH treatment. The management of growth in patients with short stature and early puberty is a frequent challenge for pediatricians. A final tall height for children is a desired outcome, and the use of combined GnRHa and rhGH treatment to increase FAH has been attempted in early puberty that does not fit the criteria for ICPP [11–13]. However, it is unclear whether such an approach is appropriate or efficacious. The primary outcomes of the previously published studies appeared different in different studies. The primary outcomes of the difference in predicted adult height (PAH) before and after therapy, the difference between PAH before therapy and FAH, the difference between PAH after therapy and target height, and GV, have all been used. However, the change in height does not adequately demonstrate the final therapeutic effect. Instead, it is preferable to assess the effect of increasing the FAH, rather than an earlier height. After the combined treatment, the BA of some of the girls advanced more quickly, so the FAH was not significantly improved. Thus, the use of FAH is preferable to determine the efficacy of combination therapy.

The aim of this meta-analysis was to evaluate the effects of GnRHa and rhGH on FAH in girls with ICPP or early puberty. Height standard deviation score (HSDS) can eliminate the effects of differing standard deviations in different age, ethnicity, and sex groups and can more accurately reflect height; thus, it was chosen as the evaluation criteria in this analysis. The use of the difference between the initial HSDS and FAH standard deviation score (FAHSDS) is preferable to determine the efficacy of combination therapy.

## 2. Materials and Methods

### 2.1. Search Strategy and Study Selection

The Medline, Cochrane, EMBASE, and Google Scholar databases were searched for studies published up to 15 March 2018 using the key words “gonadotropin releasing hormone agonist/analogue,” “precocious puberty/early puberty,” “randomized controlled trial” and “height.” The reference lists of the studies identified in this way were also examined for relevancy. Two authors independently assessed the abstracts of the studies identified by the searches for their concordance with the inclusion criteria. Studies were excluded if they were case reports, letters, comments, or editorials, or if no quantitative outcomes were reported.

The inclusion criteria for the meta-analysis are as follows: the study type is a randomized controlled trial or a quasi-randomized controlled trial; the participants are girls with early puberty or precocious puberty; and the interventions are GnRHa combined with rhGH versus GnRHa alone, rhGH alone, or no treatment. This article only included studies in which the subjects were followed up until they reached their FAH. Girls with early puberty were defined as follows: (1) chronological age at least 8 years; (2) early puberty stage (Tanner stages II–III), with GnRHa-stimulating test results indicative of central puberty [14]; and (3) slow growth or BA advancement, with unfused hand and wrist epiphyses, and height < −1 SDS. Girls with precocious puberty were defined as follows: (1) having ICPP diagnosed according to the published criteria [15]; (2) showing BA advancement with unfused hand and wrist epiphyses; and (3) having a normal serum level of thyroid-stimulating hormone. The exclusion criteria are as follows: (1) presence of a known chronic disease, whether it is being treated or not, that might influence growth rate; (2) presence of Turner syndrome or another chromosomal abnormality; or (3) an existing diagnosis of GH deficiency. The criteria for assessing FAH are BA ≥ 15 years and/or a growth rate < 1 cm/year.

### 2.2. Data Extraction and Management

A description of the details of the studies included is given in Tables 1 and 2. The following details were recorded in each study: (1) study name; (2) sample size and age; (3) intervention and control groups, study duration, type, and drug concentration, dose, and frequency; and (4) type of outcome measure (height (cm) and HSDS) or FAH and FAHSDS. HSDS was calculated as (measured height − mean height)/SD, where measured height is the actual height of a child, and the mean height and SD are the mean height and standard deviation for girls of a corresponding age. The primary outcome was the difference between the FAHSDS and the initial HSDS.

The primary researcher was requested to provide any missing data from the trials that were included, and an explanation is given where data were not provided. The review will be updated with new information when this becomes available. Review Manager software, developed by the Cochrane Collaboration, was used for data organizing and analysis (RevMan5), and data were reported as mean differences with 95% confidence intervals (CIs).

Two review authors selected trials using a simple contingency form, according to the Cochrane Collaboration checklist, to assess the risk of bias. The quality of the studies was divided into three categories using the following: (1) low risk of bias, if all six criteria were fully met; (2) unclear risk of bias, if one or more criteria were partially met; and (3) high risk of bias, if one or more criteria were not met. Clinical heterogeneity was assessed using the chi-square and *I*^2^ tests. A random-effect model was used for highly heterogeneous data (*I*^2^ > 50%, *P* < 0.1), and a fixed effects model was used for homogeneous data. The reasons for any heterogeneity were explored using subgroup analysis. All the outcome analyses were assessed for their sensitivity using the leave-one-out approach.

## 3. Results

### 3.1. Results of the Electronic Searches

**Figure 1:**
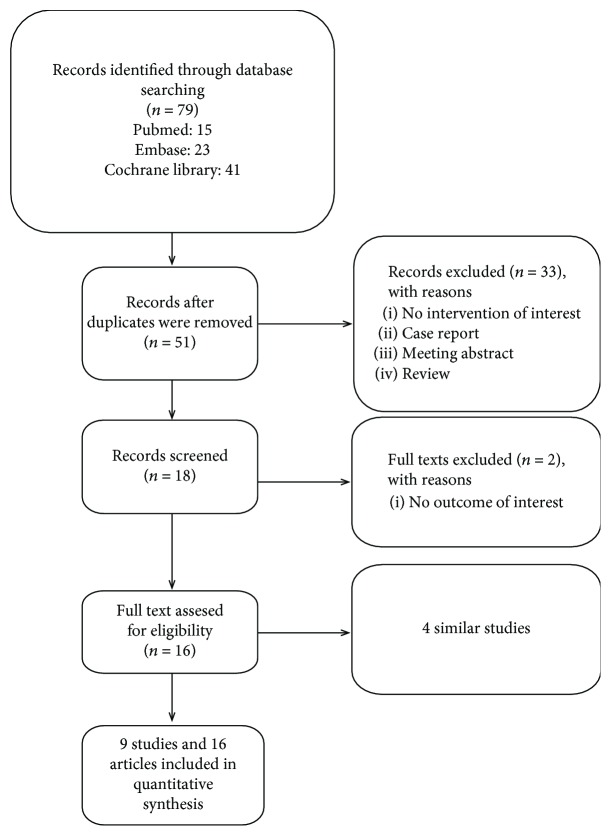
Flow diagram describing the literature searches undertaken.

The electronic searches identified 79 records. After removing duplicates, 51 records remained, and after examination of the titles and abstracts of these papers, we eliminated any studies that clearly did not match our inclusion criteria. We obtained full text copies of the 16 potentially eligible studies and subjected these to further evaluation. Nine studies were found to be eligible and included in the meta-analysis; five were studies of early puberty and four were studies of ICPP. We excluded 23 studies because they were animal experiments, reported the same study, or did not describe an intervention that fitted the selection criteria. A further two studies were excluded from the analysis because no outcomes of FAHSDS or initial HSDS were available (Tatò et al. [16], Yanovski et al. [17]) (Figure 1).

### 3.2. Description of the Studies Included in the Meta-Analysis

The nine studies included data for 304 girls and three boys. The age range of the girls with early puberty (Tanner 2–3) was under 14 years old, which included those with ISS, those who were SGA, and otherwise normal shorter children who did not meet diagnostic criteria of short stature. The age range of the girls with precocious puberty was 2–10 years old. No significant adverse effects were reported during the treatment period and after discontinuation of the therapy, including effects on glucose metabolism, thyroid dysfunction, body mass index increase, and negative effects on bone mineral density. Details of the studies included are given in Table 1 (girls with ICPP) and Table 2 (girls with early puberty) [7, 8, 9, 11–13, 18–27].

**Figure 2:**
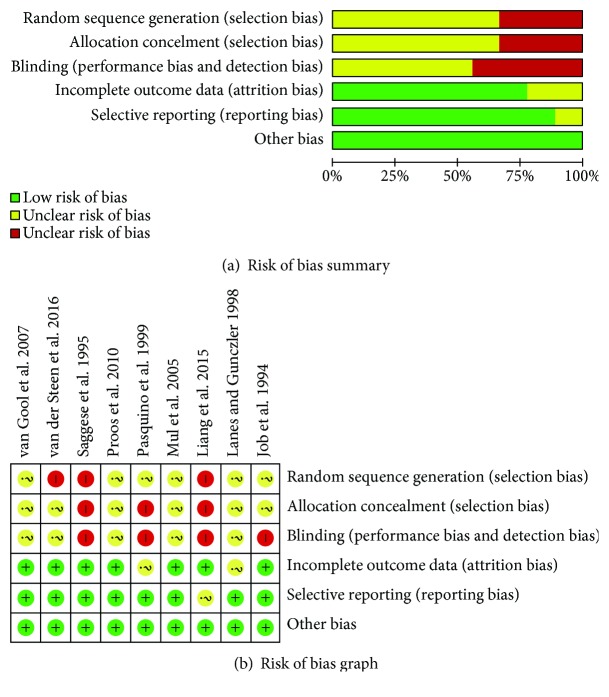
Assessment of the risk of bias in the analyzed studies. (a) The quality assessment for each included study, given as a “risk of bias” summary. (b) Outcomes are presented as percentages across all the included studies, each depicted as a “risk of bias” graph.

Tuvemo et al. [19], Tuvemo et al. [9], and Proos et al. [23, 24] report data from the same study, as do Kamp et al. [20], van Gool et al. [13], Mul et al. [8, 22], and van der Steen et al. [26, 27]. The studies of Mul et al. [22], van Gool et al. [13], Proos et al. [24], and van der Steen et al. [27], which followed the subjects through to adulthood, were analyzed. The studies of Pasquino et al. [7], Mul et al. [22], Proos et al. [24], and Liang et al. [25] were of ICPP, and the interventions compared were a GnRHa and rhGH combination and GnRHa alone. The studies of Job et al. [11], Saggese et al. [18], Lanes and Gunczler [12], van Gool et al. [13], and van der Steen et al. [27] were of early puberty. The interventions compared in Job et al. [11] and van der Steen et al. [27] were GnRHa combined with rhGH and rhGH alone, while those in Saggese et al. [18], Lanes and Gunczler [12], and van Gool et al. [13] were GnRHa combined with rhGH and no treatment. We only analyzed data from girls, except in the case of Lanes and Gunczler's study [12] that included three boys, and all of the participants reached their FAH, except for those reported in Job et al. [11] which reached their PAH. In addition, Job et al. [11] reported that HSDS decreased from −2.8 to −8.1 after 3 years of treatment (a somewhat unlikely response), and in this instance only the data from the girls were included. These biases were reduced by using strict case definitions and minimum quality criteria for the studies that were included, as shown in Figure 2. This sensitivity analysis showed that the overall result of the analysis was robust.

### 3.3. Effects of the Interventions

#### 3.3.1. The Difference between FAHSDS and Initial HSDS for Early Puberty (GnRHa and rhGH versus rhGH Alone)

**Figure 3:**
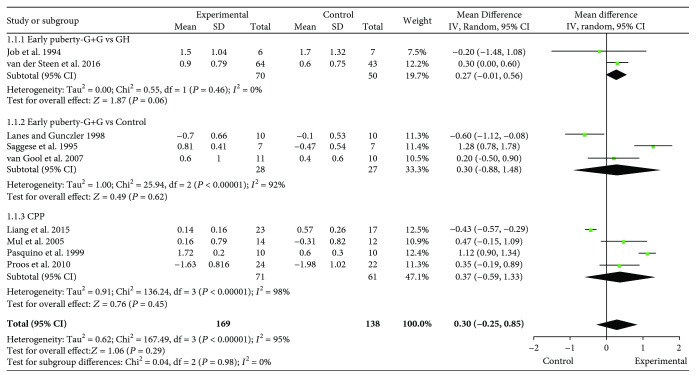
Forest plots showing the results of the meta-analysis. Forest plots showing results for the meta-analysis of (1.1.1) the difference between final adult height standard deviation score (SDS) and initial height SDS for the combined gonadotropin-releasing hormone analogs (GnRHa) and recombinant human growth hormone (rhGH) group and the rhGH alone group in girls with early puberty; (1.1.2) the difference between the final adult height SDS and initial height SDS for the combined GnRHa and rhGH group and the no treatment group in girls with early puberty; (1.1.3) the difference between final adult height SDS and initial height SDS for the combined GnRHa and rhGH group and the GnRHa alone group for girls with idiopathic central precocious puberty (ICPP). CI: confidence interval.

Because the Job et al. [11] study did not follow their participants until they achieved adult height, no FAHSDS was calculated, and we used HSDS at the end of the study *in lieu*. There was no significant heterogeneity in the two studies of early puberty (heterogeneity test: *Q* = 0.06, degrees of freedom (df) = 1 (*P* = 0.81); *I*^2^ = 0%); therefore, a fixed-effect model was used for the analysis. The overall analysis showed that the difference between FAHSDS and initial HSDS in the combined test cohorts was 0.29 SDS, which was not significantly different from that of the combined control groups (95% CI: 0–0.46, *Z* = 1.87, *P* = 0.06) (Job et al. [11], van der Steen et al. [27]) (Figure 3).

#### 3.3.2. The Difference between FAHSDS and Initial HSDS for Early Puberty (GnRHa and rhGH versus No Treatment)

**Figure 4:**
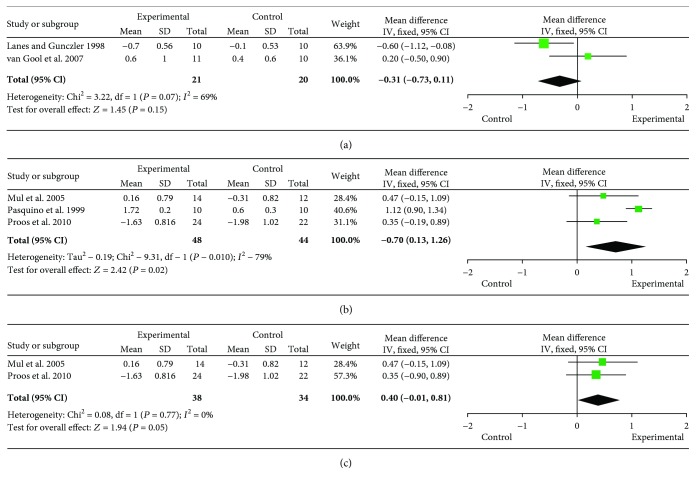
Forest plots showing the primary outcomes for girls with early puberty or CPP. Forest plots showing (a) the primary outcomes for girls with early puberty after sensitivity analysis. The difference between final adult height standard deviation score (SDS) and initial height SDS for the combination therapy versus no therapy; (b) the primary outcomes for girls with central precocious puberty (CPP) after sensitivity analysis. The difference between final adult height SDS and initial height SDS for the combination therapy versus GnRHa therapy; (c) the primary outcomes for adopted girls with idiopathic CPP after sensitivity analysis. The difference between final adult height SDS and initial height SDS for the combination therapy versus GnRHa therapy.

There was significant heterogeneity when data from the three studies of early puberty were pooled (heterogeneity test: *Q* = 25.94, df = 2, *P* < 0.00001, *I*^2^ = 92%) (Saggese et al. [18], Lanes and Gunczler [12], van Gool et al. [13]) (Figure 3). Therefore, a sensitivity analysis was conducted. The removal of the analysis of Saggese et al. [18], which contained participants who were relatively short healthy children, had a large effect, because the participants in the other two studies were small for gestational age (SGA) infants or had ISS; therefore, the data from these other studies were pooled (heterogeneity test: *Q* = 3.22, df = 1, *P* = 0.07, *I*^2^ = 69%). The overall analysis showed that the difference between FAHSDS and initial HSDS in the test cohorts was 0.31 SDS lower than that in the control cohorts (95% CI = −0.73–0.11, *Z* = 1.45, *P* = 0.15), although this did not reach statistical significance (Job et al. [11], van der Steen et al. [27]) (Figure 4(a)).

#### 3.3.3. The Difference between FAHSDS and Initial HSDS for ICPP

There was significant heterogeneity in the pooled data from the four studies of ICPP (heterogeneity test: *Q* = 136.24, df = 1, *P* < 0.00001, *I*^2^ = 98%) (Pasquino et al. [7], Mul et al. [22], Proos et al. [24], Liang et al. [25]) (Figure 3). Therefore, a sensitivity analysis was conducted. The removal of the study of Liang et al. [25], which appears to contain inaccurate data, had a large effect. This previous study concluded that the effect in group B was greater than group A; FAH in groups A and B was reported to be 159.81 cm and 161.01 cm, respectively. However, the data also showed that initial HSDS was −0.73 and FAHSDS was −0.57 in group B, while initial HSDS was −0.5 and FAHSDS was 0.07 in group A, implying that the effect in group A was greater than group B. The HSDS data were not consistent with regard to the country of origin and ethnicity of the participants, and the study was retrospective; therefore, it was excluded from the current analysis. Therefore, data from three studies were pooled (heterogeneity test: *Q* = 9.31, df = 2, *P* = 0.01, *I*^2^ = 79%) and a random-effect model of analysis was used (Figure 4(b)). There was also a large effect if the study of Pasquino et al. [7] was removed, in which the participants had not been adopted, in contrast to the participants of the other two studies, who had been adopted from developing countries. When data from these two studies of ICPP were pooled (heterogeneity test: *Q* = 0.08, df = 1, *P* = 0.77, *I*^2^ = 0%), the overall analysis showed that the difference between FAHSDS and initial HSDS in the experimental cohorts was 0.4 SDS higher than in the control cohorts (95% CI = −0.01–0.81, *Z* = 1.94, *P* = 0.05), a borderline significant result (Figure 4(c)).

## 4. Discussion

Idiopathic central precocious puberty leads to a short final stature compared with the PAH or target height, as does early puberty with advanced BA [28]. Consequently, deciding how best to improve the FAH is a common challenge for pediatricians. GnRHa is the gold standard treatment for ICPP; however, in some ICPP patients, the decrease in growth rate is marked enough for PAH to be impaired [29]. It has therefore been suggested that rhGH should be added to GnRHa therapy to increase FAH; however, previous studies have generated contrasting conclusions. Some short patients, such as those who are SGA or have ISS, are prescribed rhGH to increase their height, but they also show advanced BA at the onset of puberty, so some pediatricians add GnRHa to their treatment regimen to improve growth further [11, 27]. However, previous studies had contrasting findings. Therefore, this meta-analysis was conducted to determine whether combined GnRHa and rhGH therapy has any advantage over GnRHa alone, rhGH alone, or no treatment, for these girls, to guide future clinical treatment.

For girls with ICPP, there was no significant difference between initial HSDS and FAHSDS in this meta-analysis of three studies, probably because they may have already exhibited a higher initial HSDS because of accelerated growth. The progression of bone maturation in turn can shorten their growing period and result in tall children, but short adults [2]. Thus, it would be difficult for the combined therapy to improve FAHSDS effectively. Many factors can affect height, especially parental height. Participants in the studies of Proos et al. [24] and Mul et al. [22] were girls adopted from developing countries, in which the onset of puberty is often considerably later than in the countries the children had migrated to, resulting in lower adult height. When they were adopted, HSDS was 2 SDS less than for children of the same age in a developed country. Afterwards, catch-up growth occurred because of good nutrition, which likely advances the development of reproductive glands [22, 24]. Some of these girls had ICPP and required treatment with GnRHa, but the addition of rhGH did not increase their FAH. Therefore, we do not recommend that rhGH is added to GnRHa in the treatment of ICPP in such patients with catch-up growth. However, participants in the Pasquino et al. [7] study were girls who had not been adopted and had ICPP, and therefore did not undergo catch-up growth before diagnosis. In this situation, the use of combined therapy was associated with a significant 1.12 SDS increase in FAHSDS over the use of GnRHa alone. Thus, we recommend that such patients take rhGH in addition to GnRHa for the treatment of ICPP, if the duration of treatment with rhGH can be at least 2 years, although the quoted effect may be one of rhGH alone, and further studies are required to assess these possibilities.

For girls with ISS or SGA, catch-up growth occurs after rhGH treatment (Lanes and Gunczler [12], van Gool et al. [13]), but BA advances quickly when they enter puberty, and the addition of GnRHa to rhGH cannot influence FAHSDS. The increase in HSDS achieved through the use of combined therapy was limited in SGA. Furthermore, the effect of GnRHa to delay BA was lost when the combined treatment regimen ended. In fact, BA advanced more rapidly, such that this expensive treatment did not generate a better outcome, but rather a worse one. Therefore, we do not recommend the use of the combined therapy instead of rhGH treatment to increase the FAH of girls with ISS or SGA.

For short, healthy girls who do not undergo catch-up growth before taking the combined therapy, FAH significantly increases afterwards (Saggese et al. [18]). At least 2 years of combined therapy can increase HSDS by 1.28 over no treatment when FAH is reached. This effect seems similar to the effect of rhGH described above, and the combined treatment may be recommended for these patients if additional future studies demonstrate consistent findings. However, the participants in the studies of Job et al. and van der Steen et al., like in the studies of Lanes and Gunczler and van Gool et al., were girls with ISS or SGA, and no significant effect of the combined therapy was demonstrated on FSHSDS versus rhGH alone. Therefore, we do not recommend the use of the combined therapy versus no treatment to increase the FAH of girls with ISS or SGA.

Three meta-analyses have been conducted in this area in the last 10 years. Li et al. suggested that GnRHa therapy may have a positive effect on PAH in girls with early puberty and that the addition of rhGH to the treatment regimen may confer an advantage [30]. However, the authors included patients with both EP and ICPP who were not followed to their FAH. For girls with ICPP, the GnRHa and rhGH combination therapy can improve FAH by a mean of 2.81 cm over GnRHa alone as reported by Liu et al. [31]. Bertelloni et al. indicated that GnRHa treatment does not significantly change the growth outcome in girls with EP [32]. It is believed that the use of the combined therapy for precocious puberty and early puberty is capable of resulting in a greater FAH, and indeed the findings of the present analysis are not ideal. The reason for the limited improvement shown may be the time of treatment, or heredity may be a decisive factor.

Pharmacoeconomics must also be considered when Chinese girls are treated for ICPP. The weight of a pubertal girl is ~40 kg, and the combined treatment takes 2 years. 50–100 *μ*g/kg/day of rhGH are required, and therefore the treatment is 2–4 mg/month. The price of this much rhGH is ~1178 China Yuan (CNY)/10 mg. Therefore, the entire cost is CNY 84,816–113,088 for 2 years. Importantly, medical insurance does not cover such a cost in China, meaning that families must spend a large amount of money themselves.

## 5. Conclusions

The overall analysis of the data fails to indicate any benefit of combined therapy, while the individual reports suggest that in specific instances combined therapy may be beneficial in preserving or reclaiming growth potential and improving adult height. The following conclusions are derived: (1) The increase in HSDS achieved through the use of the combined therapy is limited in SGA and catch-up growth girls with ICPP. They may achieve an approximately 2 cm increase in FAH if their parents are willing to pay for 2 years of this expensive therapy. (2) The combined therapy significantly increases FAHSDS by 1.12 SDS over the use of GnRHa alone in nonadopted girls with ICPP. (3) For normal shorter children, the combined therapy seems very effective, with FAHSDS being increased by 1.28 SDS in which the effect of rhGH was not excluded. (4) However, for ISS, the combined therapy seems even worse than rhGH alone or no treatment.

## Figures and Tables

**Table 1:** Characteristics of included studies of girls with CPP.

Authors (year)	Comparison	Treatment	Duration of therapy	Subjects (*n*)	Before treatment					After treatment	
					CA (years)	BA (years)	Height (cm)	HSDS	PAH (cm)	FAH (cm)	FAHSDS
Pasquino et al. 1999 [7]	GnRHa	Trip 100 *μ*g/kg/21 days, i.m.	2–4 yr.	10 Italy	7.6 ± 0.2	10.4 ± 0.3	NA	−1.0 ± 0.3	155.5 ± 2.0	157.1 ± 2.5	−0.4 ± 0.3
	GnRHa + GH	Trip + GH 0.3 mg/kg/wk, sc.		10 Italy	10.0 ± 0.5	12.0 ± 0.2		−1.5 ± 0.2	152.7 ± 1.7	160.6 ± 1.3	0.2 ± 0.2
Proos et al. 2010 [24]	GnRHa	Buserelin 0.3 mg/4 wk, sc. implant	2–4 yr.	22 adopted	8.2 ± 0.8	9.7 ± 1.1	130.0 ± 7.4	0.3 ± 1.0	163.6 ± 6.5	155.8 ± 6.9	−1.6 ± 1.1
	GnRHa + GH	Buserelin + GH 0.1 U/kg/day, sc.		24 adopted	8.4 ± 0.8	10.2 ± 0.6	132.3 ± 5.6	0.5 ± 1.1	163.1 ± 5.6	158.9 ± 5.4	−1.1 ± 0.9
Mul et al. 2005 [22]	GnRHa	Trip 3.75 mg/28 days, i.m.	3 yr.	12 adopted	9.6 ± 0.9	10.7 ± 1.1	133.8 ± 8.7	−2.3 ± 0.6	149.8 ± 5.6	155.0 ± 5.6	−2.1 ± 0.9
	GnRHa + GH	Trip + GH 1.33 mg/m^2^/day, sc.		14 adopted	9.6 ± 0.9	11.6 ± 0.8	135.1 ± 5.7	−1.8 ± 0.7	146.8 ± 4.8	155.0 ± 5.5	−2.1 ± 0.9
Liang et al. 2015 [25]	GnRHa	Trip 3.75 mg/28 days, sc.	8 yr.	17 China	8.1 ± 0.2	9.2 ± 0.3	132.8 ± 1.6	−0.5 ± 0.1	161.6 ± 0.9	159.8 ± 1.2	0.1 ± 0.3
	GnRHa + GH	Trip + GH 0.15–0.175 *μ*g/kg/day, sc.		23 China	7.6 ± 0.3	8.4 ± 0.3	126.6 ± 1.8	−0.7 ± 0.2	160.0 ± 1.0	161.0 ± 1.0	−0.6 ± 0.2

**Table 2:** Characteristics of included studies of girls with early puberty.

Authors (year)	Comparison	Treatment	Duration of therapy	Subjects (*n*)	Before treatment					After treatment	
					CA (years)	BA (years)	Height (cm)	HSDS	PAH (cm)	FAH (cm)	FAHSDS
Job et al. 1994 [11]	GH	GH 0.1 IU/kg/day, sc.	3 yr.	7 France ISS	12.2 ± 1.5	10.4 ± 0.8	131.4 ± 6.4	−2.8 ± 0.5	148.7 ± 8.3	152.8 ± 6.4	−1.1 ± 1.5
	GnRHa + GH	GH + trip 3.75 mg/28 days, i.m.		6 France ISS	12.3 ± 0.7	10.5 ± 0.7	133.3 ± 6.8	−2.8 ± 0.5	150.8 ± 9.2	154.6 ± 8.4	−1.3 ± 1.2
Saggese et al. 1995 [18]	Control	No treatment	2.0 ± 0.5 yr.	7 Italy short children	11.3 ± 0.6	11.0 ± 0.8	NA	−1.5 ± 0.7	149.0 ± 4.5	152.0 ± 3.4	−1.7 ± 0.5
	GnRHa + GH	Trip 60 *μ*g/kg/28 days + GH 0.65 IU/kg/wk, sc.		7 Italy short children	11.5 ± 0.9	11.5 ± 0.8		−1.8 ± 0.6	146.8 ± 4.4	156.1 ± 2.1	−0.9 ± 0.3
Lanes and Gunczler 1998 [12]	Control	No treatment	2.5 yr.	10 USA ISS	11.4 ± 1.0	11.0 ± 0.8	128.9 ± 7.8	−2.3 ± 0.4	151.8 ± 10.1	150.3 ± 9.8	−2.9 ± 0.8
	GnRHa + GH	Leup 0.3 mg/kg/28 days, i.m. + GH 0.1 U/kg/day, sc.		10 USA ISS	11.8 ± 1.3	11.2 ± 0.9	128.9 ± 7.5	−2.4 ± 0.4	150.7 ± 9.8	151.7 ± 2.4	−2.7 ± 0.6
van Gool et al. 2007 [13]	Control	No treatment	3 yr.	15 Netherlands SGA/ISS	11.8 ± 0.7	NA	136.1 ± 4.5	−2.5 ± 0.5	160.0 ± 10.1	159.5 ± 5.7	−2.3 ± 0.6
	GnRHa + GH	GH 0.14 IU/kg/day, sc. + trip 3.75 mg/28 days, i.m.		17 Netherlands SGA/ISS	11.6 ± 0.7		135.4 ± 4.5	−2.4 ± 0.5	157.4 ± 8.3	161.8 ± 6.3	−2.0 ± 1.0
van der Steen et al. 2016 [27]	GH	GH 1–2 mg/m^2^/day, sc.	5.9 yr.	43 Dutch SGA	7.6 ± 3.2	NA	NA	−2.3 ± 0.7	NA	NA	−1.7 ± 0.8
	GnRHa + GH	Leup 3.75 mg/kg/28 days, sc. + GH		64 Dutch SGA	7.3 ± 3.1			−2.8 ± 0.6			−1.8 ± 0.9

Trip is triptorelin. i.m. is intramuscularly. Leup is leuprolide. sc. is subcutaneous.
